# Two Negative-Strand RNA Viruses Identified in Watermelon Represent a Novel Clade in the Order Bunyavirales

**DOI:** 10.3389/fmicb.2017.01514

**Published:** 2017-08-09

**Authors:** Min Xin, Mengji Cao, Wenwen Liu, Yingdang Ren, Xueping Zhou, Xifeng Wang

**Affiliations:** ^1^State Key Laboratory for Biology of Plant Diseases and Insect Pests, Institute of Plant Protection, Chinese Academy of Agricultural Sciences Beijing, China; ^2^National Citrus Engineering Research Center, Citrus Research Institute, Southwest University Chongqing, China; ^3^Institute of Plant Protection, Henan Academy of Agricultural Sciences Zhengzhou, China

**Keywords:** watermelon (*Citrullus lanatus*), Next Generation Sequencing (NGS), negative-sense single-stranded RNA virus, novel virus clade, *Bunyavirales*

## Abstract

Two novel negative-sense, single-stranded (ss) RNA viruses were identified in watermelon plants and named watermelon crinkle leaf-associated virus 1 and 2 (WCLaV-1 and -2), respectively. The multipartite genomes consist of three RNA molecules of ~6.8, 1.4, and 1.3 kb. The genomes and the deduced proteins of RNA1 and RNA3 show features resembling those of members in the genus *Phlebovirus* and *Tenuivirus*; however, the predicted proteins encoded by RNA2 are related to the movement protein (MP) in the genus *Ophiovirus* and *Emaravirus*. Furthermore, these two viruses define a novel clade in the family *Phenuiviridae*, order *Bunyavirales*, which is phylogenetically related to the viruses in the above four genera. Moreover, after mechanical inoculation with WCLaV-1 seedlings of the natural host watermelon plants develop crinkling similar to those observed in the field. These findings enhance our understanding of the evolution and the classification of ssRNA viruses.

## Introduction

Watermelon (*Citrullus lanatus*) is an economically important fruit crop in many countries and is nutritionally beneficial to humans (Weng and Sun, 2012). In open fields, watermelon plants are frequently exposed to viruses such as watermelon mosaic virus (WMV), zucchini yellow mosaic virus (ZYMV), cucumber mosaic virus (CMV), cucumber green mottle mosaic virus (CGMMV), tobacco mosaic virus (TMV), and squash mosaic virus (SqMV) that can cause great losses in production (Liu et al., 2009). In recent years, novel viruses that infect watermelon have been discovered through the use of next-generation sequencing (NGS). For example, *Citrullus lanatus* cryptic virus (CiLCV), a putative member of the genus *Deltapartitivirus* in the family *Partitiviridae* and two orthotospoviruses, watermelon bud necrosis virus (WBNV), and melon severe mosaic virus (MeSMV) in the family *Tospoviridae* have been detected in watermelon plants (Ciuffo et al., 2009; Li et al., 2011; Sela et al., 2013; Xin et al., 2017a).

In the tenth Report of the International Committee on Taxonomy of Viruses (ICTV), the *Bunyaviridae* Study Group of the International Committee on Taxonomy of Viruses (ICTV) established the new order *Bunyavirales* to accommodate nine families that comprise 13 genera (https://talk.ictvonline.org/taxonomy/). These viruses are a unique group that infect a wide range of hosts, including vertebrates, invertebrates, and plants (Webster et al., 2011). Three of the families (genera) contain members that have plants as their primary hosts: *Fimoviridae* (*Emaravirus*), *Phenuiviridae* (*Tenuivirus*), and *Tospoviridae* (*Orthotospovirus*). Members of the order *Bunyavirales* have similarities in genome organization and particle morphology, e.g., spherical in emaraviruses and orthotospoviruses and filamentous in tenuiviruses (Falk and Tsai, 1998; Mielke-Ehret and Mühlbach, 2012; Zhang et al., 2016). Their genomes consist of three to eight segments of linear negative-sense (or ambisense, depending on the genus) RNA, e.g., large (L), medium (M), and small (S), which encode the viral RNA polymerase, two glycoproteins as a single gene product that is usually co-translationally cleaved, and the nucleocapsid protein, respectively (Elliott and Blakqori, 2011; Léger and Lozach, 2015). The nucleotide sequences at the 3′-terminus and the 5′-terminus of each segment are complementary, resulting in RNAs that form panhandle structures (Kormelink et al., 2011; Jackson and Li, 2016).

In 2015 and 2016, during a field survey in Kāifēng, Hénán Province, China, virus-like symptoms on leaves of watermelon were observed. To identify potential causal agents, RNA sequencing (RNA-seq) and small RNA sequencing (sRNA-seq) were carried out. Here, we describe and discuss the molecular and biological properties of two new (-ss) RNA viruses that were discovered from the infected leaves, named watermelon crinkle leaf-associated viruses 1 and 2 (WCLaV-1 and -2), as possible representatives of a novel taxon in the family *Phenuiviridae*, order *Bunyavirales*.

## Materials and methods

### Sample collection, small RNA sequencing, and RNA sequencing

In July 2015, leaf samples with mosaic and curling symptoms were collected from watermelon plants of cv. Hēixiùlóngjǔanfēng grown in the field in Hénán Province, China. Subsequently, in June 2016, watermelon samples with virus-like symptoms were randomly collected from Kāifēng County, Hénán Province. Samples were stored at −70°C.

Symptomatic leaves from cv. Hēixiùlóngjǔanfēng (sample KF-1) and healthy leaves (sample KF-0) were selected for small RNA-sequencing (sRNA-seq) in 2015; leaves coded KF-15 were used for RNA-sequencing (RNA-seq) in 2016. Total RNAs were extracted using TRIzol reagent (Invitrogen, Carlsbad, CA, USA) following the manufacturer's protocol but modified to repeat the chloroform extraction and subsequent centrifugation step (Liu et al., 2015). A BioAnalyzer 2100 (Agilent Technology, Santa Clara, CA, USA) was used to assess the quality of the purified RNA, then the purified RNA was quantified using a Nanodrop ND-2000 spectrophotometer (Infinigen Biotechnology, City of Industry, CA, USA). The Ribo-Zero Magnetic Kit (Plant Leaf) (Epicentre, Madison, WI, USA) was used to deplete the ribosomal RNA from transcriptomes for RNA-seq, then the library was constructed with a TruSeq RNA Sample Prep Kit (Illumina, San Diego, CA, USA), and a TruSeq Small RNA Sample Prep Kit (Illumina) was used to construct the sRNA libraries. Subsequently, the Illumina Hiseq2500 platform (1 × 50 bp read lengths) was used for sRNA-seq (SinoGenoMax, Beijing, China), and the Hiseq 4000 platform was used for RNA-seq with PE150 bp (Biomarker Biology Technology, Beijing, China). Raw reads from the Illumina platform were trimmed of adaptor sequences and low-quality reads by the CLC Genomics Workbench 9.5 (Qiagen, Valencia, CA, USA). The Velvet program was used for *de novo* assembly with a *k*-mer of 17 for small RNA data (Zerbino and Birney, 2008; Wu et al., 2010). The CLC Genomics Workbench 9.5 was used for *de novo* assembly for RNA-seq data. The assembled contigs were subsequently screened against the NCBI databases using a BLASTn and BLASTx search with standard parameters.

### Amplification of full genomes

To verify the presence of viruses in field samples, we used RT-PCR and specific primers designed on the sequences of assembled contigs (Table S4). The sequences of the extreme ends of the genomic RNAs were determined employing the 5′- and 3′-RACE System for Rapid Amplification of cDNA Ends kits (Thermo Fisher Scientific, Waltham, MA, USA) (Than et al., 2016). The primers and other oligonucleotides used for RT-PCR and RACE analyses are listed in Table S3. The PCR products were separated by electrophoresis in 1.0% agarose gels and visualized by ethidium bromide staining, then purified with the Wizard SV Gel and PCR Clean-Up System (Promega, Madison, WI, USA). The final products were cloned into the PEASY-T5 cloning vector (TransGen Biotech, Beijing, China), and then inserted into Trans-T1 Chemically Competent Cells (TransGen). At least eight clones from each subclone were sequenced (Beijing Genomics Institute, Beijing, China).

### Sequence and phylogenetic analyses

Sequences were analyzed and assembled using Vector NTI 11.5, then submitted to the GenBank database in NCBI using the software Sequin. The reconstructed genome was subjected to standard sequence analyses: (I) prediction of ORFs using the ORF Finder program in NCBI (http://www.ncbi.nlm.nih.gov/projects/gorf/); (II) identification of conserved and functional domains of the predicted proteins in WCLaV-1 and WCLaV-2 using the Conserved Domain Database (CDD) in NCBI (https://www.ncbi.nlm.nih.gov/Structure/cdd/cdd.shtml) (Marchler-Bauer et al., 2015) and the SMART tool (http://smart.embl-heidelberg.de/) (Letunic et al., 2015); analyses of the core RdRp and NP domains in other (-ss) RNA viruses using the CDD; (III) multiple sequence alignment of the core motifs in RdRp and the conserved nucleotide sequence at the 5′- and 3′-end by CLC Genomics Workbench 9.5; (IV) identity analyses using the needle program in WebLab (http://weblab.cbi.pku.edu.cn/); (V) multiple sequence alignments first using the Clustal W method and then MEGA 6.0 (Tamura et al., 2013) for phylogenetic tree construction using the neighbor-joining method with 1,000 bootstrap replicates, the Poisson model and pairwise gap deletion options. Abbreviations and accession numbers of viruses used in this study are listed in Table S5.

### Mechanical transmission in a controlled environment

The crude sap, extracted from leaves (about 1 g) infected by WCLaV-1 and homogenized in 10 mL 0.01 M phosphate buffer (PB) (pH = 7.2), was used for mechanical transmission tests on four-leaf-stage seedlings of watermelon cv. Hēixiùlóngjǔanfēng. Plants non-inoculated and inoculated only by PB were used as negative controls. After inoculation, the seedlings were maintained in a clean growth chamber at 25°C and 80% relative humidity with 16-h light/8-h dark. The mechanically inoculated plants were carefully observed for symptoms and subsequently tested for virus infection by RT-PCR assays with the virus-specific primer pairs listed in Table S4 and sequenced at 17, 42, and 67 days post inoculation (dpi). The mechanical transmission experiments were repeated three times with 20, 23, and 46 healthy watermelon plants inoculated, respectively. The cDNA was synthesized using Moloney murine leukemia virus (MoMLV) reverse transcriptase (Promega), and the PCR protocol employed 32 cycles after an initial denaturation at 95°C for 5 min. Each cycle consisted of denaturation at 95°C for 45 s, primer annealing for 45 s at 55°C and an extension for 1 min at 72°C, followed by an elongation step of 10 min at 72°C. The products were separated using 1.0% agarose gel electrophoresis.

## Results

### Identification of two unique negative-stranded RNA viruses of watermelon using next-generation sequencing

**Figure 1:**
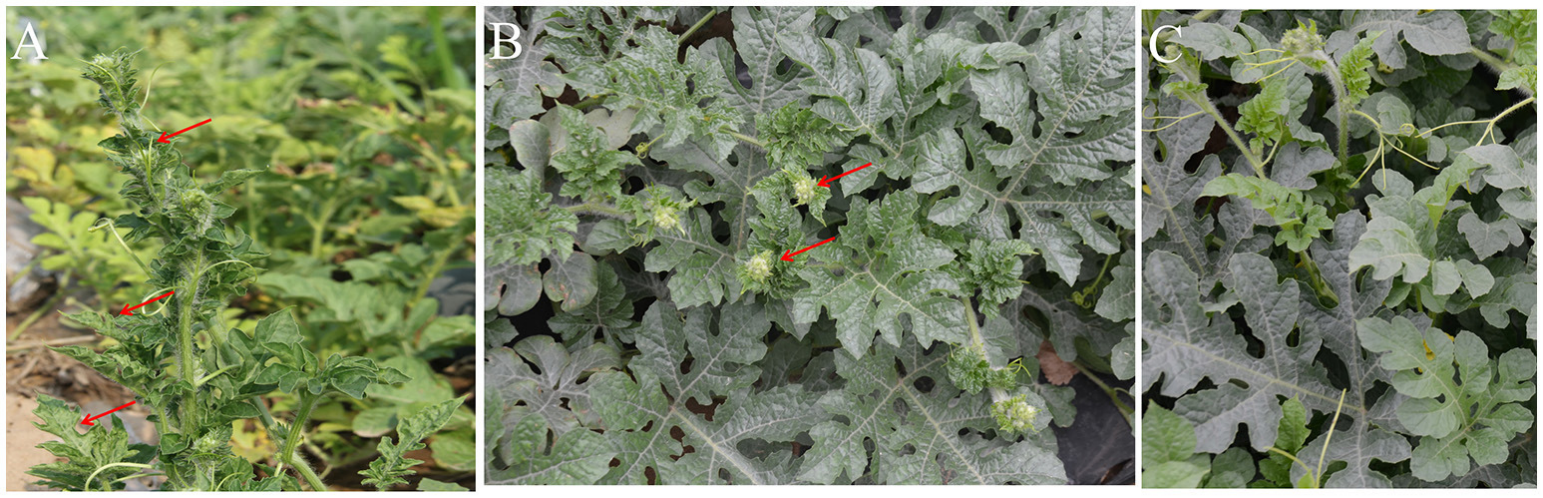
Symptoms on WCLaV-1- and WCLaV-2-infected watermelon leaves in the field **(A)** sample KF-1, crinkling, and mosaic, on the leaves of watermelon plants used for small RNA-sequencing for WCLaV-1 in 2015; **(B)** sample KF-15, cluster/crinkling on the top of leaves used for RNA-sequencing for WCLaV-2 in 2016; **(C)** symptomless leaves from uninfected watermelon plants. Red arrows in **(A,B)** indicate the crinkle leaf symptom.

Our field investigation revealed diseased plants of watermelon with various virus-like symptoms, including leaf crinkling, mosaic, and bunchy top (Figures 1A,B); no symptoms were observed on the seemingly healthy plants (Figure 1C). To identify the viruses present in symptomatic samples, we subjected samples KF-1 and KF-15 to sRNA-seq and RNA-seq, respectively. Healthy plant KF-0 was also small RNA-sequenced. For sRNA-seq, a total of 22,235,704, and 20,667,689 reads with length between 18 and 27 nucleotides (nt) were obtained in sample KF-0 and KF-1, respectively. After *de novo* assembly and BlastX and BlastN searches against nr and nt databases, ZYMV (genus *Potyvirus*), WMV (genus *Potyvirus*), melon aphid-borne yellows virus (MABYV, genus *Polerovirus*), CiLCV (genus *Deltapartitivirus*), and four contigs homologous to viruses in the genera *Phlebovirus* and *Ophiovirus* were identified in sample KF-1 (Table S1). In contrast, contigs from KF-0 did not show any significant similarity with previously reported viruses. For RNA-seq, a total of 16,547,389 reads with a length of 150 nt were obtained after filtering the reads mapping to the watermelon genome (Guo et al., 2013). Interestingly, we identified CiLCV, CGMMV (genus *Tobamovirus*), watermelon virus A (WVA, family *Betaflexiviridae*), and three contigs homologous (high similarity with that identified in KF-1) to phleboviruses and ophioviruses in the sample KF-15 collected in 2016 (Table S1). Reverse transcription-polymerase chain reaction (RT-PCR) using specific primers (Table S4) and sequencing of the amplified products confirmed the presence of these contigs.

### Genomic organization of WCLaV-1 and WCLaV-2

**Figure 2:**
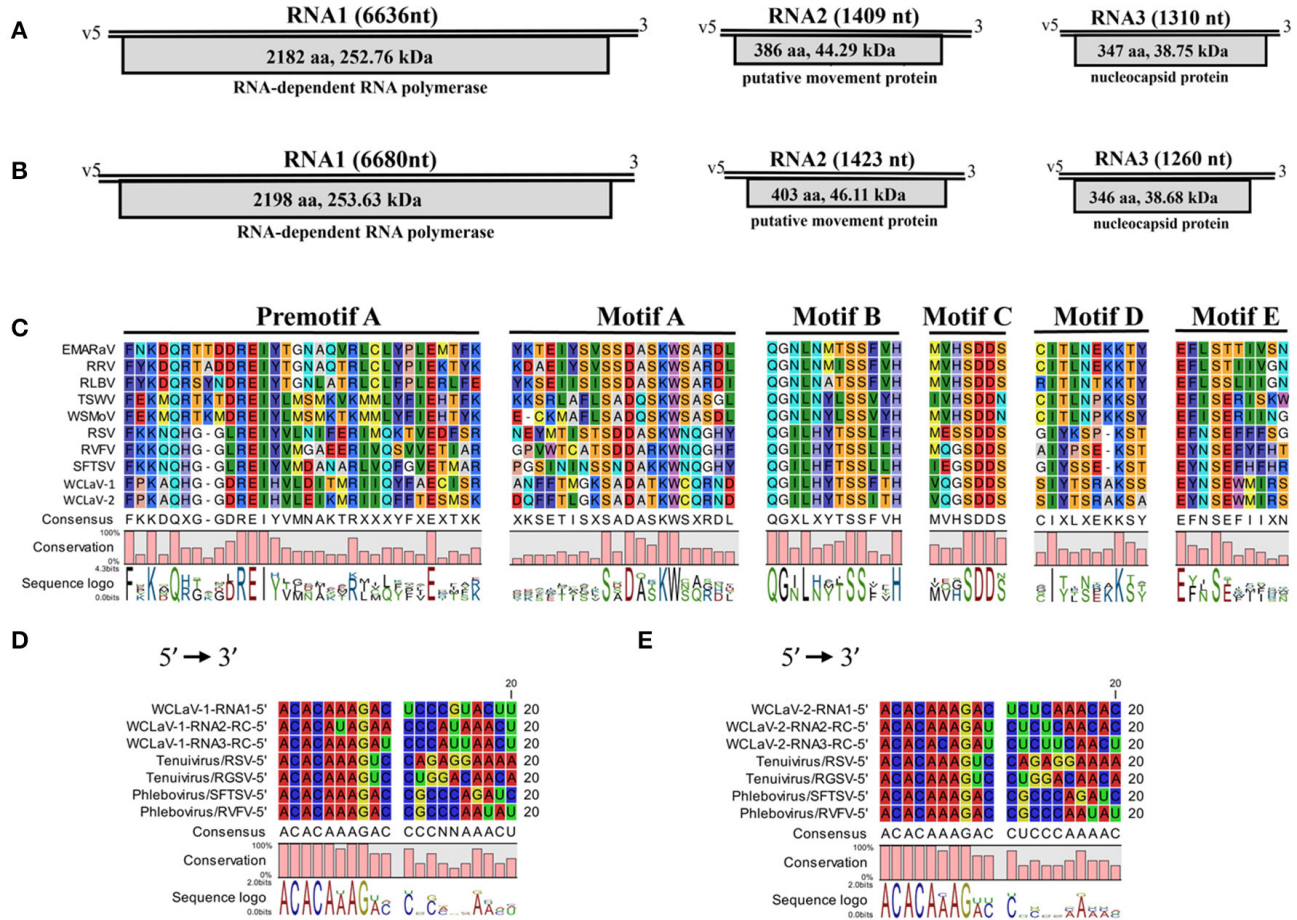
Schematic representation of the genome organizations of WCLaV-1 **(A)** and WCLaV-2 **(B)**. Open reading frames (ORFs) and deduced products of each RNA are shown as gray boxes, with the amino acid (aa) length, estimated molecular weight (kDa), and function of the putative proteins. **(C)** Amino acid alignment between conserved RdRp premotif A and motifs A–E of WCLaV-1, WCLaV-2, and selected (-ss) RNA viruses. The conserved nucleotide stretches are present at the termini of WCLaV-1 **(D)** or WCLaV-2 **(E)** with tenuiviruses and phleboviruses. RC, reverse and complementary sequence; nt, nucleotides; v, viral RNA; EMARaV, European mountain ash ringspot-associated virus (AY563040); RRV, rose rosette virus (HQ871942); RLBV, raspberry leaf blotch virus (FR823299); TSWV, tomato spotted wilt virus (D10066); WSMoV, watermelon silver mottle virus (AF133128); RSV, rice stripe virus (D31879); RVFV, Rift Valley fever virus (DQ375403.1); SFTSV, severe fever with thrombocytopenia syndrome (SFTS) virus (HM745930); RGSV, rice grassy stunt virus (AB009656).

The genomes were obtained by RT-PCR and RACE-PCR (Figure S1) using virus-specific primers designed on the sequences of these contigs (Tables S3, S4). The assembled genomes are composed of three RNA segments, with complementary-sense RNAs (vcRNAs) of these two viruses predicted to contain a single ORF (Figures 2A,B). The nucleotide sequences reported here have been deposited in GenBank (respectively, accession KY781184, KY781185, KY781186 for RNA1, RNA2, and RNA3 of WCLaV-1; KY781187, KY781188, KY781189 for RNA1, RNA2, and RNA3 of WCLaV-2).

RNA1 for both viruses is the largest segment and has only one ORF (ORF1): 6,636 nt and 6,680 nt for WCLaV-1 and -2, which start, respectively, at position 6,584–6,586 and 6,634–6,636 and end at position 38–40 and 40–42. ORF1 encodes a putative RNA-dependent RNA polymerase (RdRp, p1) of 2,182 amino acids (aa) and 2,198 aa with a predicted molecular weight of 252.76 and 253.63 kDa, respectively, for WCLaV-1 and -2 (Figures 2A,B). A Blastp search with the algorithm PSI-BLAST using the deduced aa sequences of p1 show that they have about 25% identity to the L proteins of viruses in the genus *Phlebovirus* (Table S2). Additionally, SMART analyses recognized the conserved domain Bunya_RdRp (Pfame-value: 5.7e-51, 1.1e-47) in the p1 protein of these two viruses (Table S2). Interestingly, the conserved domain RRM_SF super family (RNA recognition motif superfamily, also known as RNA binding domain) was predicted by the Conserved Domain Database for p1 in WCLaV-2 (interval: 1,386–1,443; *E*-value: 4.22e-04), but was not found in WCLaV-1 (Marchler-Bauer et al., 2015). The alignment of RdRp of WCLaV-1, WCLaV-2, and other (-ss) RNA viruses indicates that the amino acid sequences of their RdRps contain the six conserved motifs (premotif A and motifs A–E), which represent highly conserved regions of the RdRp of the members in the order *Bunyavirales* (Figure 2C). Both WCLaV-1 and WCLaV-2 have the motif A (DATKWC), motif B (QGILHYTSS), and motif D (KS). Motif C includes the SDD sequence. The motif E tetrapeptide E(F/Y)xS, which is specifically conserved in the polymerases of segmented negative-sense RNA viruses (van Poelwijk et al., 1997; Kormelink et al., 2011), was also found in the proteins encoded by RNA1 of these two viruses (Figure 2C). In addition, we identified three basic residues (K, R, and R/K) in premotif A and a glutamic acid (E) downstream of premotif A (Figure 2C), which are also conserved in bunyavirus RdRps (Bruenn, 2003; Elbeaino et al., 2009). Moreover, the endonuclease domains H_66_-D_78_-PD_95−96_-E_107_-K_126_, similar to that in phlebo- and tenuiviruses and differing from the H-D-PD-DxK-T in orthotospoviruses, were also observed in the N-terminus of WCLaV-1 and WCLaV-2. These domains are probably involved in cap-snatching, a genome expression strategy to cap viral mRNAs proposed for members of the previous family *Bunyaviridae* and genus *Tenuivirus* (Biswas and Nayak, 1994; Reguera et al., 2010). In addition, the core RdRp domain of WCLaV-1 and WCLaV-2 is closely related to that of the tenuiviruses and phleboviruses, respectively; WCLaV-1 shares 27.6% aa sequence identity with Salehabad virus (SALV, genus *Phlebovirus*) and WCLaV-2 shares 27.2% aa sequence identity with rice stripe virus (RSV, genus *Tenuivirus*), both remarkably higher than with viruses in other genera, but far lower than the 75% identity between these two viruses.

RNA2 of WCLaV-1 (1,409 nt) and WCLaV-2 (1,423 nt) may, respectively, encode a protein (p2) of 44.29 and 46.11 kDa, spanning position 1,356–1,358 and 1,366–1,368 (AUG start codon) to position 196–198 and 155–157 (UGA stop codon) of vcRNA (Figures 2A,B). Interestingly, stretches highly rich in U were found in the 5′-terminus of RNA2 of both viruses. Blastp searches showed that the deduced aa sequences of RNA2 have 24% identity (28/116; *E* = 1.4) with the 54 kDa protein of citrus psorosis virus (CPsV, genus *Ophiovirus*), demonstrating a putative movement protein (MP) function, for WCLaV-1 and 19% identity (25/133; *E* = 1.4) to the 58 kDa MP of blueberry mosaic associated virus (BIMaV) in the genus *Ophiovirus* (Robles Luna et al., 2013; Borniego et al., 2016) for WCLaV-2 (Table S1). Therefore, the deduced protein p2 of WCLaV-1 and WCLaV-2 may be the putative MP. Nevertheless, no significant conserved domains in p2 of these two viruses have been identified using CDD and SMART analyses (Table S2). Pairwise comparisons showed that the aa sequence identities between the p2 of WCLaV-1, WCLaV-2 and other ssRNA viruses ranged, respectively, from 2.2 to 18.3% and 9.7 to 18.5%; the highest aa identities, 18.3 and 18.5%, respectively, were found with proteins encoded by RNA2 of groundnut bud necrosis virus (GBNV, the genus *Orthotospovirus*) (Table 1). Compared with the aa identity of 46.1% between these two viruses, the identities with known viruses are very low. Altogether, these data revealed that p2 encoded by RNA2 of these two viruses has unique features worth further study.

**Table 1:** Amino acid sequences identities between WCLaV-1, WCLaV-2, and other (-ss) RNA viruses.

**Genus**	**Virus abbreviation**	**Amino acid identities (%)**									
		**RdRp**		**Core RdRp**		**Putative MP**		**NP**		**Core NP**	
		**WCLaV-1**	**WCLaV-2**	**WCLaV-1**	**WCLaV-2**	**WCLaV-1**	**WCLaV-2**	**WCLaV-1**	**WCLaV-2**	**WCLaV-1**	**WCLaV-2**
*Emaravirus*	EMARaV	17.6	17.4	19.0	17.4	11.1	9.7	14.3	14.7	–	–
	RRV	17.6	16.6	18.7	17.6	16.7	13.8	9.2	12.0	–	–
	RLBV	17.5	16.6	17.7	18.8	2.2	16.9	10.9	16.0	–	–
	HPWMoW	17.1	17.3	17.4	17.0	17.6	17.2	15.3	13.3	–	–
*Tenuivirus*	RSV	17.8	18.6	25.8	**27.2**	14.6	17.1	15.5	14.1	19.6	17.3
	RGSV	17.8	18.0	26.8	26.5	17.1	16.4	16.8	15.4	**21.2**	13.0
*Phlebovirus*	RVFV	20.2	20.9	25.5	24.4	–	–	14.0	14.1	19.7	19.0
	SFTSV	21.2	22.1	25.6	24.5	–	–	16.1	15.5	19.2	16.7
	UUKV	20.9	20.8	26.8	25.9	–	–	17.0	16.0	20.5	20.2
	SFNV	21.8^*^	22.1	27.5	26.5	–	–	16.6	**17.4**	20.3	21.5
	PuTV	21.5	22.1	26.8	27.1	–	–	16.3	18.7	21.0	**23.2**
	SALV	21.2	**22.7**	**27.6**	26.9	–	–	**17.2**	17.3	19.8	20.8
	CDUV	20.9	22.1	26.3	25.5	–	–	13.5	14.9	16.9	20.4
	BUJV	20.7	21.9	27.3	26.9	–	–	15.7	15.4	19.6	19.7
*Orthotospovirus*	TSWV	16.7	16.4	15.8	16.2	13.4	11.2	13.7	13.7	16.8	15.2
	WBNV	15.2	17.4	17.2	16.1	15.6	12.8	5.8	10.2	7.6	3.1
	WSMoV	15.7	17.4	17.3	16.1	14.0	15.8	5.4	12.7	4.3	13.4
	INSV	16.3	16.3	16.9	16.1	14.7	15.1	13.4	6.0	16.0	4.3
	GBNV	15.9	17.2	17.1	16.2	**18.3**	**18.5**	3.2	9.7	4.0	4.1
	IYSV	16.9	15.6	17.3	16.6	16.9	11.2	16.1	4.6	16.1	7.8
	PolRSV	17.0	15.2	16.7	16.2	17.0	11.4	10.9	12.2	13.5	12.0
*Orthohantavirus*	HTAV	15.6	17.3	15.1	17.0	–	–	14.7	4.4	8.1	2.7
*Orthobunyavirus*	BUNV	17.1	17.5	17.7	17.1	–	–	5.8	5.2	0.7	0.8
*Orthonairovirus*	CCHFV	12.1	11.8	16.9	17.5	–	–	11.2	7.5	0.8	1.2
*Ophiovirus*	CPsV	17.5	17.2	–	–	13.6	12.2	16.9	13.0	10.2	2.8
	LRNV	13.6	14.1	–	–	10.7	16.5	15.0	12.5	6.7	15.7
	BIMaV	15.6	15.6	–	–	13.0	13.5	10.4	13.2	17.4	18.7
	MLBVV	15.2	16.2	–	–	14.3	13.2	16.8	10.5	17.4	3.7
	WCLaV-1	–	**59.0**	–	**75.0**	–	**46.1**	–	**47.9**	–	**56.8**
	WCLaV-2	59.0^**^	–	**75.0**	–	**46.1**	–	**47.9**	–	**56.8**	–

* *Numbers in red represent highest identities of WCLaV-1 and WCLaV-2 with selected (-ss) RNA viruses*.

** *Numbers reported in green represent WCLaV-1 and -2 mutual identities*.

^***^The extended names of viruses corresponding to virus abbreviations are shown in Table S5.

RNA3 of WCLaV-1 is 1,310 nt long and 1,260 nt for WCLaV-2. The ORFs start at position 1,251 and 1,198 nt, respectively, with an AUG start codon and terminate with an UAA stop codon at position 208 and 158 nt, respectively, putatively encoding a nucleoprotein (NP, p3) of 347 a and 346 aa with a molecular mass of 38.75 and 38.68 kDa, respectively (Figures 2A,B). It is worth noting that U-rich regions are also observed at the 5′-terminus in RNA3 of these two viruses, similar to the case in RNA2. According to the CDD and SMART analyses, RNA3 encodes proteins that contain the conserved domains Tenui_N super family (accession cl05345), the typical domains in nucleocapsid proteins of viruses in the genera *Tenuivirus* and *Phlebovirus*, with a Pfame-value of 5.4D-16, 3.2e-17, respectively, distinct from those in other genera. Moreover, the highest amino acid identities for the nucleocapsid protein, 17.2 and 17.4%, respectively, were found with SALV and sandfly fever Naples virus (SFNV, genus *Phlebovirus*) for WCLaV-1 and WCLaV-2, far lower than the 47.9% aa identity between these two viruses for nucleocapsid protein (Table 1). They share the highest aa identities, 21.2 and 23.2%, respectively, with the core NP domains of rice grassy stunt virus (RGSV, genus *Tenuivirus*) for WCLaV-1 and Punta Toro virus (PTV, genus *Phlebovirus*) for WCLaV-2 (Table 1).

In addition, the highly conserved nucleotides 5′-ACACAAAG-3′ at the 5′-terminus were found in each RNA1 segment, and the conserved nucleotides 3′-UGUGUUUC-5′ at the 3′-terminus were detected in each RNA2 and RNA3 segment (Figures 2D,E). The 5′- and 3′-terminal regions exhibited obvious complementarity, which is a common characteristic of genomic segments in tenuiviruses and phleboviruses (Elliott and Blakqori, 2011).

### Phylogenetic relationships of the WCLaV-1 and WCLaV-2 with other (-ss) RNA viruses

**Figure 3:**
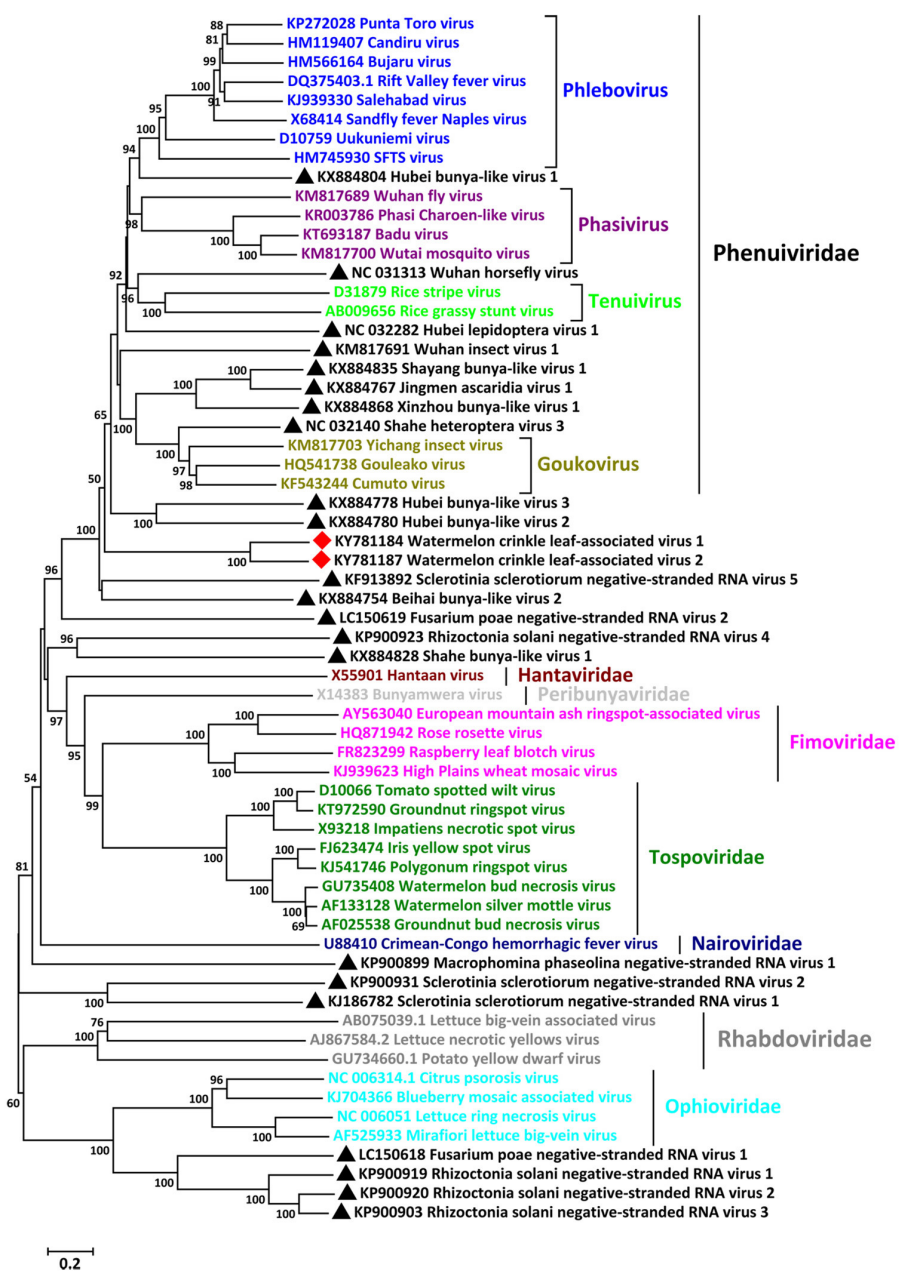
Phylogenetic analysis of WCLaV-1, WCLaV-2 based on the amino acid sequences of putative RdRp, and the corresponding proteins of representative negative-sense RNA viruses. The tree was generated by the neighbor-joining method using the Poisson model, and bootstrap values were estimated using 1,000 replicates and MEGA 6.0. The scale bar represents number of substitutions per amino acid site. Viruses in the same genera or family are labeled with the same colors. Viruses found in this study are marked with red squares; unclassified viruses are marked with black triangles.

To reveal the relationships of WCLaV-1 and WCLaV-2 with known (-ss) RNA viruses, we constructed phylogenetic trees based on the amino acid sequences of the putative RdRp (Figure 3), MP (Figure S2A) and NP (Figure S2B). According to the generated trees based on amino acid sequences of RdRp, WCLaV-1, and WCLaV-2 are most closely related to viruses in the genera *Tenuivirus, Phlebovirus, Goukovirus, Phasivirus*, the family *Phenuiviridae* (Figure 3). However, phylogenetic analyses based on the amino acid sequences of RNA2-encoded proteins indicate these two viruses consistently cluster with emaraviruses and ophioviruses (Figure S2A). WCLaV-1 and WCLaV-2 form a distant and separate clade with the selected (-ss) viruses in all the phylograms, revealing that they may belong to a novel genus.

### Mechanical transmission of WCLaV-1 in a controlled environment

**Figure 4:**
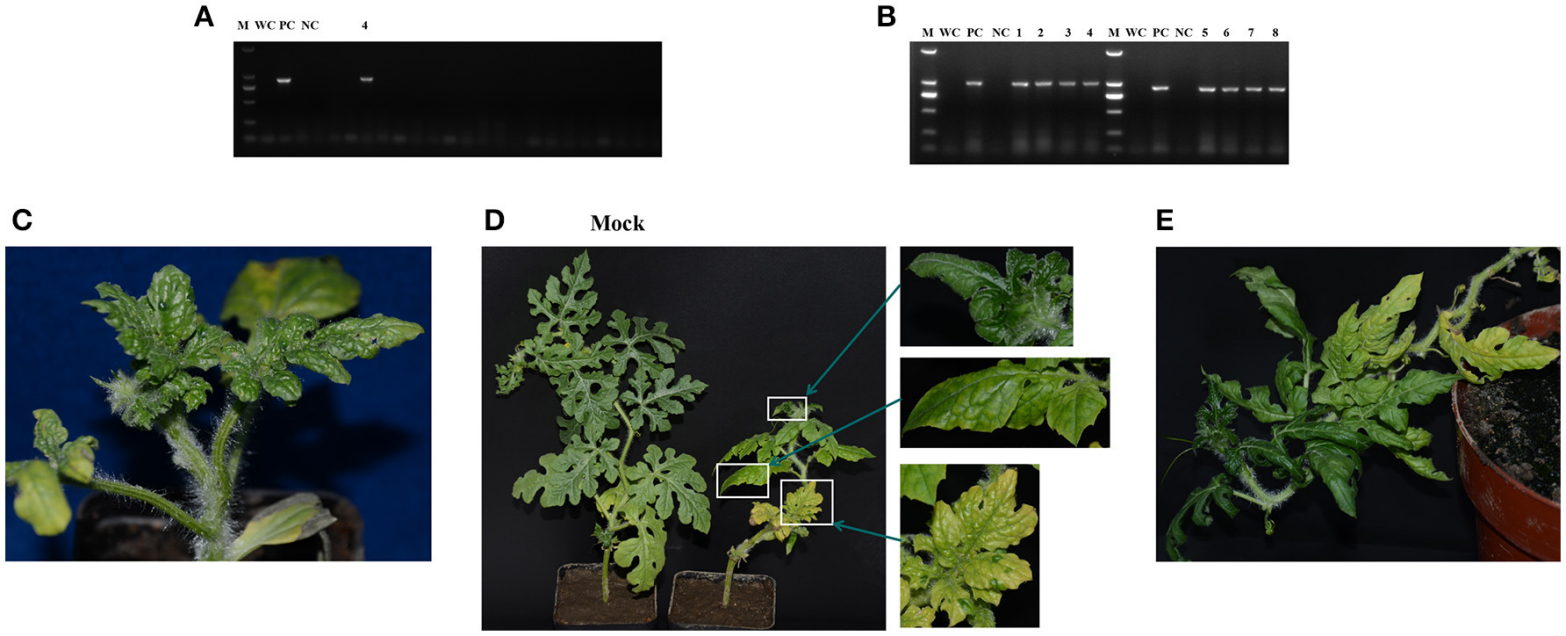
Test for mechanical transmission of watermelon crinkle leaf-associated virus 1 (WCLaV-1) to watermelon. **(A,B)** RT-PCR and agarose gel electrophoresis to detect infected watermelon leaves. **(A)** At 17 days post-inoculation (dpi), only sample No. 4 was infected; **(B)** detection of systematic infection in four leaves of sample No. 4 using two primer pairs in RT-PCR. Symptom development over time **(C–E)**. **(C)** Crinkling at 17 dpi; **(D)** mosaic and stunt at 42 dpi compared with the non-inoculated plant and close ups of symptoms; **(E)** curling and mosaic at 67 dpi. M, DNA Marker DL2000 (Takara); PC, positive control; NC, uninfected watermelon control; WC, water control.

The mechanical transmission experiment with WCLaV-1 was done three times with 20, 23, and 46 healthy watermelon plants inoculated, respectively. Each time, only one plant, respectively, of the inoculated samples was positive for WCLaV-1 infection by RT-PCR, thus conclusively showing infection rates of 5.0, 4.3, and 2.2%, respectively. For example, at 17 dpi, WCLaV-1 was detected only in watermelon plant No. 4 using RT-PCR with primer pair NP-1F/R, and the positive band was further identified by sequencing (Figure 4A, Table S4). Moreover, four uninoculated leaves of watermelon plant No. 4 were confirmed as infected by WCLaV-1 by RT-PCR detection with primer pairs NP-1F/R and MP-1F/R (Figure 4B), thus demonstrating that the watermelon plant was systemically infected by WCLaV-1. WCLaV-1 was not detected in the control plants non-inoculated and inoculated with phosphate buffer (PB). By 17 dpi, new leaves of watermelon seedlings had developed obvious mosaic and crinkling similar to the symptoms observed on plants in the field (Figure 4C). Subsequently, at 42 dpi, watermelon plant No. 4 displayed obvious crinkling and dwarf symptoms, which were lacking in the non-inoculated plant (Figure 4D). At 67 dpi, leaf crinkling was also observed (Figure 4E).

## Discussion

The development of NGS and bioinformatics has accelerated the discovery of novel viruses (Adams et al., 2009; Hadidi et al., 2016; Massart et al., 2017). Unlike traditional techniques such as ELISA, PCR, or hybridization for diagnosing viruses, NGS technology requires no prior knowledge of the host or viral nucleotide sequences because virus-specific siRNAs are generated during an antiviral defense response in the plants infected by any virus, and the virus-specific siRNAs overlap in sequence, and thus can be assembled into long, contiguous fragments (contigs) of the invading viral genome (Al Rwahnih et al., 2009; Kreuze et al., 2009; Cao et al., 2014) With this technology, many viruses with negative-sense single-stranded (ss) RNA genomes, such as BIMaV and pigeonpea sterility mosaic virus 2 (PPSMV-2) have been characterized (Elbeaino et al., 2015; Li et al., 2015; Patil and Kumar, 2015). Indeed, our group previously used this technology to characterize the novel plant virus watermelon virus A (Xin et al., 2017b) and CiLCV, infecting watermelon (Xin et al., 2017a). In this study, we discovered and characterized two novel (-ss) RNA viruses from watermelon using NGS.

These two novel viruses share many common features with viruses in the newly established order *Bunyavirales. First* of all, the conserved sequences SDD in motif C, the signature of the core domain in RdRp for the segmented negative-stranded RNA viruses in the families *Orthomyxoviridae, Arenaviridae*, and the previous *Bunyaviridae* (now *Peribunyaviridae*), as opposed to the GDNQ characteristic of non-segmented negative-stranded RNA viruses, are found in the putative RdRp of these two viruses (Kormelink et al., 2011). Likewise, the sequence E(F/Y)xS in motif E, which is specifically conserved in the polymerases of segmented (-) RNA viruses (Müller et al., 1994; Reguera et al., 2010), were also discovered, demonstrating that these two viruses are segmented (-ss) RNA virus. Furthermore, the eight terminal conserved bases are identical to the corresponding regions in the genomes of phleboviruses and tenuiviruses; The deduced RdRp and NP are homologous to the corresponding proteins of bunyaviruses, sharing higher sequence identities and possessing the same conserved domains with viruses in the genera *Phlebovirus* and *Tenuivirus* (Kormelink et al., 2011). Phylogenetic analyses of viral RdRp and NP consistently place these two viruses in the bunyavirus supergroup, most closely related to, but distinct from phleboviruses and tenuiviruses (Elliott and Blakqori, 2011; Mielke-Ehret and Mühlbach, 2012). These observations suggest an evolutionary link between these two viruses with tenuiviruses and phleboviruses, distinguished from ophioviruses and emaraviruses.

Although WCLaV-1 and WCLaV-2 share sequence similarities with the members of the genera *Tenuivirus, Phlebovirus, Ophiovirus*, and *Emaravirus*, they have some unique properties and are markedly distant from all of the viruses in the above four genera. First, genomes of most (-ss) RNA viruses (except ophioviruses) generally contain a glycoprotein (GP) precursor, which has not been detected in WCLaV-1 and WCLaV-2 so far. Second, no conserved domains were found in the proteins encoded by RNA2, predicted as the MPs of WCLaV-1 and WCLaV-2, which are widely found in plant-infecting (-ss) RNA viruses (Kormelink et al., 2011). Third, all of the aa identities with the representative (-ss) RNA viruses in RNA1–3 are far lower than those between WCLaV-1 and WCLaV-2, e.g., the highest aa identity (22.7%) with known viruses was lower than the aa identity of 59.0% between these two viruses in RNA1. Fourth, they did not form clades that included any of the selected (-ss) RNA viruses; in contrast, these two viruses clustered on the same branch in the phylogenetic trees. Fifth, the genomes of all emaraviruses consist of four or more negative-sense RNAs, with the fully conserved 13-nt sequences in the 5′- and 3′-termini. Further, the longest RNA segments possess two ORFs in the same strand, a particular property of viruses in the genus *Ophiovirus*. The RNA3 segments in tenuiviruses and phleboviruses use an ambisense strategy, encoding one protein in the 5′-region of the vRNA and another protein in the vcRNA (Kormelink et al., 2011; Mielke-Ehret and Mühlbach, 2012). Because not all these features have been found in WCLaV-1 and WCLaV-2, they cannot now be assigned to any described genus based on molecular features and phylogenetic analyses. Taken together, these data indicate that WCLaV-1 and WCLaV-2 constitute part of a new virus cluster that bridges the genera *Tenuivirus, Phlebovirus, Ophiovirus*, and *Emaravirus*.

There is a highly conserved stretch 5′-ACACAAAG-3′ in the 5′-terminal of RNA1, but it is not found in RNA2 or RNA3. In contrast, the conserved nucleotides 5′-CUAUGUGU-3′ were only observed in the 3′-terminal of RNA2 and RNA3, not in RNA1, which may also imply a longer sequence. Although attempts to identify additional WCLaV-1 and WCLaV-2 genomic fragment(s) by RT-PCR and deep analyses of virus-derived sRNAs and contigs failed, we cannot exclude the potential existence of one or more additional genomic RNAs. Additionally, the sequences may include stretches due to cap snatching because the RACE was performed on total RNA.

Questions regarding the biology of WCLaV-1 and WCLaV-2 still need to be answered. Iris yellow spot virus (genus *Orthotospovirus*, family *Tospoviridae*) can be transmitted to healthy onions by mechanical inoculation in a controlled glasshouse at a relatively low transmission rate (Bag et al., 2015). Likewise, WCLaV-1 can also be mechanically transmitted to the natural host watermelon as confirmed in this study. The low transmission rates after mechanical inoculation indicate that other factors may be involved in the transmission of these viruses. Indeed, many bunyavirales are vectored by arthropods such as mosquitoes, flies, ticks, planthoppers, and thrips, and the associated arthropods also serve as virus reservoirs in many cases (Kormelink et al., 2011; Léger and Lozach, 2015). During the field survey, a large population of thrips was found in the watermelon plants, so we will investigate thrips species in the field where the samples were collected and test them in a transmission assay. We hope to find the major vector insects of these two viruses, then explore their roles in disease epidemics.

In conclusion, we are the first to report the genomic organization, phylogenetic relationship, and biological characterization of two new (-ss) RNA viruses, which are confirmed as members belonging to a previously undescribed novel clade in the family *Phenuiviridae*, order *Bunyavirales*.

## Author contributions

XW: conceived and designed the experiments. MX, WL, YR, and MC: performed the experiments. MX, WL, XZ, and MC: analyzed the data. XW, MX, and MC: wrote the manuscript. All authors read and approved the final manuscript.

### Conflict of interest statement

The authors declare that the research was conducted in the absence of any commercial or financial relationships that could be construed as a potential conflict of interest.
