# Supplementary material for: Two Negative-Strand RNA Viruses Identified in Watermelon Represent a Novel Clade in the Order Bunyavirales
Source: Front Microbiol. 2017 Aug 9;8:1514. doi: 10.3389/fmicb.2017.01514 (PMC5552725; doi:10.3389/fmicb.2017.01514)
Supplement: Supplementary file 1 [file Presentation1.pdf]

Supplementary Materials

Two negative-strand RNA viruses identified in watermelon represent a novel clade in the order *Bunyavirales*

Min Xin, Mengji Cao*, Wenwen Liu, Yingdang Ren, Xueping Zhou, Xifeng Wang*

* **Correspondence:** Corresponding Author: xfwang@ippcaas.cn; caomengji@cric.cn

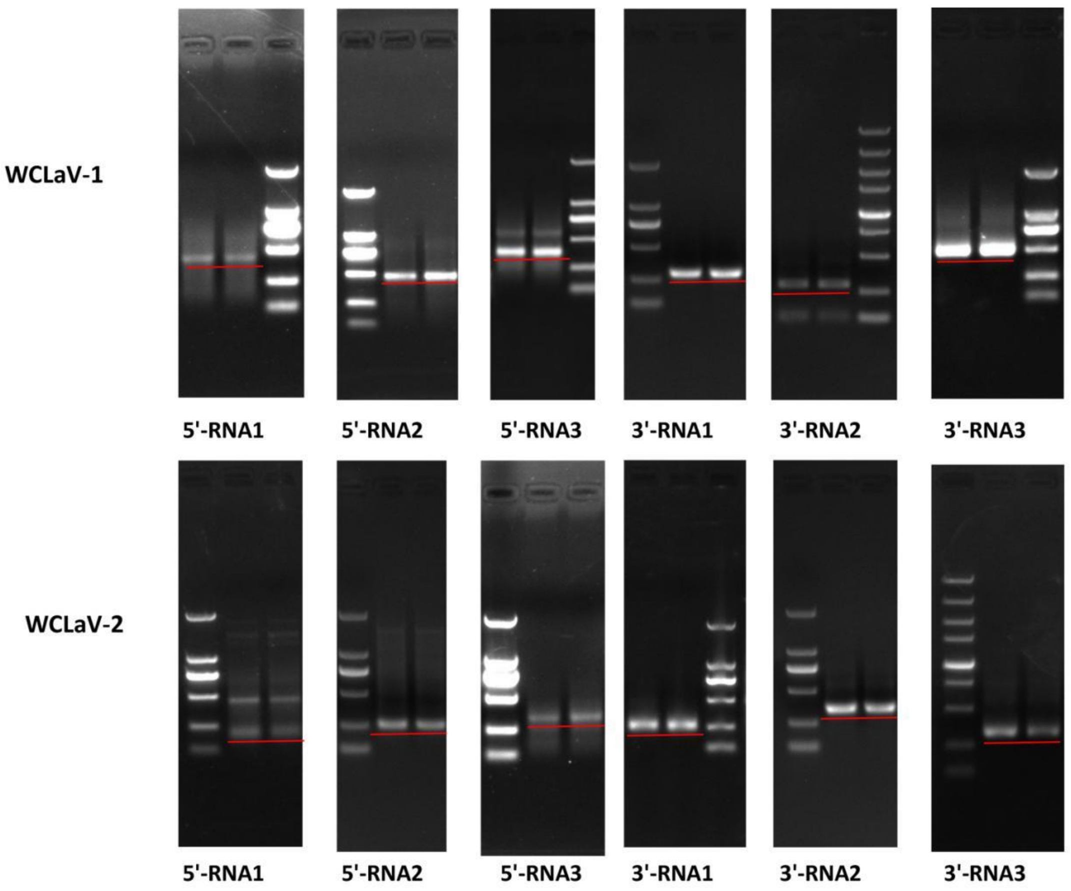

FIGURE S1 | Agarose gel electrophoresis of products from 5′ RACE and 3′ RACE of RNAs 1–3 in 1.0 % agarose gel. Target bands are marked with red lines.

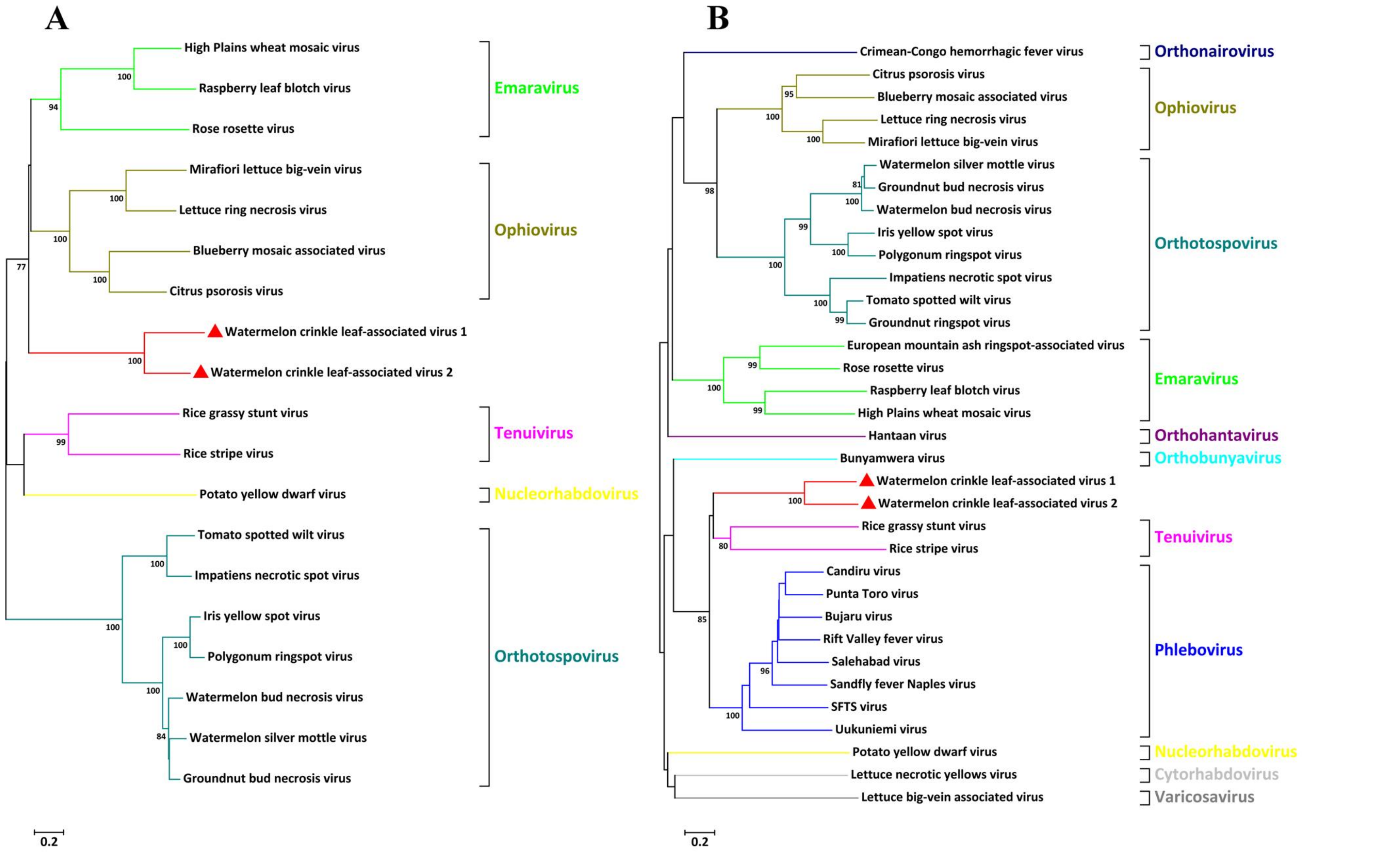
A
High Plains wheat mosaic virus
Raspberry leaf blotch virus
Rose rosette virus
Emaravirus
Mirafiori lettuce big-vein virus
Lettuce ring necrosis virus
Blueberry mosaic associated virus
Citrus psorosis virus
Ophiovirus
Watermelon crinkle leaf-associated virus 1
Watermelon crinkle leaf-associated virus 2
Rice grassy stunt virus
Rice stripe virus
Tenuivirus
Potato yellow dwarf virus
Nucleorhabdovirus
Tomato spotted wilt virus
Impatiens necrotic spot virus
Iris yellow spot virus
Polygonum ringspot virus
Watermelon bud necrosis virus
Watermelon silver mottle virus
Groundnut bud necrosis virus
Orthotospovirus
0.2
B
Crimean-Congo hemorrhagic fever virus
Orthonairovirus
Citrus psorosis virus
Blueberry mosaic associated virus
Lettuce ring necrosis virus
Mirafiori lettuce big-vein virus
Ophiovirus
Watermelon silver mottle virus
Groundnut bud necrosis virus
Watermelon bud necrosis virus
Iris yellow spot virus
Polygonum ringspot virus
Impatiens necrotic spot virus
Tomato spotted wilt virus
Groundnut ringspot virus
Orthotospovirus
European mountain ash ringspot-associated virus
Rose rosette virus
Raspberry leaf blotch virus
High Plains wheat mosaic virus
Emaravirus
Hantaan virus
Orthohantavirus
Bunyamwera virus
Orthobunyavirus
Watermelon crinkle leaf-associated virus 1
Watermelon crinkle leaf-associated virus 2
Rice grassy stunt virus
Rice stripe virus
Tenuivirus
Candiru virus
Punta Toro virus
Bujaru virus
Rift Valley fever virus
Salehabad virus
Sandfly fever Naples virus
SFTS virus
Uukuniemi virus
Phlebovirus
Potato yellow dwarf virus
Nucleorhabdovirus
Lettuce necrotic yellows virus
Cytorhabdovirus
Lettuce big-vein associated virus
Varicosavirus
0.2

FIGURE S2 | Phylogenetic analysis of WCLaV-1, WCLaV-2 based on the amino acid sequences of putative MP (A) and NP (B) with the corresponding proteins of representative negative-sense RNA viruses. The GenBank accession numbers are shown in Table S5.

TABLE S1 | Contigs obtained by *de novo* assembly of sRNAs from symptomatic watermelon samples and sharing identities with ophioviruses and phleboviruses.

	Contig	Length (nt)	BlastX results	E-value	Identities	Accession No.
**WCLaV-1**	contig 21	3886	RNA-dependent RNA polymerase [severe fever with thrombocytopenia virus]	1e-71	220/768 (29%)	AMK05809.1
	contig 94	2725	RNA-dependent RNA polymerase [Wuhan insect virus 16]	7e-41	234/889 (26%)	APG79216.1
	contig 223	1018	54 kDa protein [Citrus psorosis virus]	1.3	28/116 (24%)	AEU04873.1
	contig 86	906	nucleopasid protein [Bole Tick Virus 1]	2e-14	56/175 (32%)	AJG39301.1

	Contig	Length (nt)	BlastX results	E-value	Identities	Accession No.
**WCLaV-2**	contig_162	6660	RNA-dependent RNA polymerase [Huangpi Tick Virus 2]	5e-113	419/1688 (25%)	YP_009293590.1
	contig_961	1513	58kDa Movement protein [Blueberry mosaic associated virus]	1.8	25/133 (19%)	AJR35805.1
	contig_268	1267	nucleopasid protein [Bole Tick Virus 1]	6e-09	56/195 (29%)	AJG39301.1

TABLE S2 | Features of proteins encoded in the negative-sense orientation by WCLaV-1 and WCLaV-2 genome.

**Virus**	**Gene name**	**Open reading frames (ORF) position**	**Calculated *Mr* (KDa)**	**Isoelectric point (pI)**	**Blastp match in NCBI**	**E-value**	**Query coverage (%)**	**Identities**	**Predicted domains (SMART)**	**Amino acid position of predicted domain**
WCLaV-1	RNA1	38-6586	252.76	6.64	RNA-dependent RNA polymerase of Guertu virus, genus *Phlebovirus*, ID: APG79349.1	4e-108	61	361/1430 (25%)	Bunya_RdRp (5.7e-51)	556-1240
	RNA2	198-1358	44.29	6.39	54 kDa protein of Citrus psorosis virus, genus *Ophiovirus*, ID: AEU04874.1	1.4	29	28/116 (24%)	No significant	—
	RNA3	208-1251	38.75	8.64	nucleocapsid of sandfly fever Naples virus, Genus *Phlebovirus*, ID: ABQ23550.1	7e-11	65	58/232 (25%)	Tenui_N (5.4e-16)	92-309
WCLaV-2	RNA1	40-6636	253.63	6.20	RNA-dependent RNA polymerase of Huangpi tick virus 2, genus *Phlebovirus*, ID: YP_009293590.1	6e-117	71	421/1688 (25%)	Bunya_RdRp (1.1e-47)	553-1242
	RNA2	157-1368	46.11	7.55	58 kDa Movement protein of blueberry mosaic associated virus, genus *Ophiovirus*, ID: AJR35805.1	1.8	29	25/133 (19%)	No significant	—
	RNA3	158-1198	38.68	8.83	Nucleocapsid of sandfly fever Naples virus, genus *Phlebovirus*, ID: ABQ23550.1	2e-14	68	68/246 (28%)	Tenui_N (3.2e-17)	79-292

—: Not applicable.

TABLE S3 | List of primer sequences used to amplify the genomes of WCLaV-1 and WCLaV-2.

Virus	RNA	Primer name	Primer sequences (5'-3')	F/R	Primer position	Purpose
WCLaV-1	RNA1	RNA1-5'-GSP1	CAGATACTTGGAAAAT	R	319-334	5' primer for synthesis of 1st-stranded cDNA
		RNA1-5'-GSP2	CTGCCAAACTCTTATAGGACAA	R	268-289	5'-RACE PCR
		RNA1-5'-GSP3	TGGCTGGATACGAGACTTGTGG	R	63-84	5'-RACE Nested-PCR
		RNA1-3'-GSP2	GGCATCGGATTGAAATAACAT	F	6350-6370	3'-RACE PCR
		RNA1-3'-GSP3	CAGTAGATAAGAAAAGAGTTGG	F	6505-6526	3'-RACE Nested-PCR
		RdRp-Z- F	GGACTACCACTGAAGCAAAC	F	3709-3728	the gap between RNA1
		RdRp-Z-R	CATCCTGTAGAATTATTGCC	R	4038-4057	
	RNA2	RNA2-5'-1-GSP1	TACCCCCGTCTGTGCT	R	580-595	5' primer for synthesis of 1st-stranded cDNA
		RNA2-5'-1-GSP2	GCTCAAAGACGGAAAGTAAGA	R	510-530	5'-RACE PCR
		RNA2-5'-1-GSP3	GGAACAGACAAAGCCAATCACAT	R	440-462	5'-RACE Nested-PCR
		RNA2-5'-2-GSP1	CAAAGACGGAAAGTAAGAAAC	R	507-527	5' primer for synthesis of 1st-stranded cDNA
		RNA2-5'-2-GSP2	GGAACAGACAAAGCCAATCAC	R	442-462	5'-RACE PCR
		RNA2-5'-2-GSP3	ATTGAAGAGGAGATTACCAG	R	259-278	5'-RACE Nested-PCR
		RNA2-3'-GSP2	AACCCTTGCTCGTTTTCGG	F	1162-1180	3'-RACE PCR
		RNA2-3'-GSP3	TCCCTTCGGTTGGCTGCTGG	F	1298-1317	3'-RACE Nested-PCR
	RNA3	RNA3-5'-1-GSP1	CCCTGGAATAGTGATAC	R	630-646	5' primer for synthesis of 1st-stranded cDNA
		RNA3-5'-1-GSP2	GTCTCAAACACTCCCTGCTG	R	558-577	5'-RACE PCR
		RNA3-5'-1-GSP3	CCCTCCAGAGTCTATTCGTGTC	R	337-358	5'-RACE Nested-PCR
		RNA3-5'-2-GSP1	GATGTTGAGGGTCTGTT	R	404-420	5' primer for synthesis of 1st-stranded cDNA
		RNA3-5'-2-GSP2	TCCAGAGTCTATTCGTGTCAAA	R	334-355	5'-RACE PCR
		RNA3-5'-2-GSP3	CAGAAAACAAGAAAAGGCACAA	R	96-117	5'-RACE Nested-PCR
		RNA3-3'-GSP2	CAACAGCAGAGGGAGTCATAGC	F	1083-1104	3'-RACE PCR
		RNA3-3'-GSP3	CAATAAGTTCTGCGTTAGTGTG	F	1224-1245	3'-RACE Nested-PCR
WCLaV-2	RNA1	RNA1-5'-GSP1	GTATCACCCAGAAATG	R	495-510	5' primer for synthesis of 1st-stranded cDNA

		RNA1-5’-GSP2	GAGCATTCAACTTACAGGGA	R	194-213	5’-RACE PCR
		RNA1-5’-GSP3	TTCAAGGCTACGAGACAGGAGG	R	98-119	5’-RACE Nested-PCR
		RNA1-3’-GSP2	CAGCCTTGTCTTCATAACTAATA	F	6324-6346	3’-RACE PCR
		RNA1-3’-GSP3	TGGCTCTGGAAAATCTGAACAC	F	6541-6562	3’-RACE Nested-PCR
		RNA2-5’-GSP1	ACTCTGTTGACCCTGA	R	326-341	5’ primer for synthesis of 1st-stranded cDNA
		RNA2-5’-GSP2	AGATGAAGAAGTGGATGTTGAGG	R	219-241	5’-RACE PCR
	RNA2	RNA2-5’-GSP3	GGAAGAAAGGCTGATGAAGGG	R	185-205	5’-RACE Nested-PCR
		RNA2-3’-GSP2	GGCAACATACTGAAACCTCTT	F	1126-1146	3’-RACE PCR
		RNA2-3’-GSP3	CATCTCTTGCCACCTGCTCATT	F	1239-1260	3’-RACE Nested-PCR
		RNA3-5’-GSP1	TTACCTCCAGTGATGC	R	505-520	5’ primer for synthesis of 1st-stranded cDNA
		RNA3-5’-GSP2	TGCGTCATCAGGCTTTTGCT	R	488-507	5’-RACE PCR
	RNA3	RNA3-5’-GSP3	TTGATGATTTGCTTGTTACTGC	R	240-261	5’-RACE Nested-PCR
		RNA3-3’-GSP2	CTGCTATCCTTGCCTCTGTTATC	F	940-962	3’-RACE PCR
		RNA3-3’-GSP3	GCCGATTGTTCACTTTCAGG	F	1116-1135	3’-RACE Nested-PCR
		AAP	GGCCACGCGTCGACTAGTACGGGI IGGGIIGGGIIG	F		5’ Abridged Anchor Primer, supplied with 5’ RACE System for Rapid Amplification of cDNA Ends Kit
WCLaV-1 and WCLaV-2	RNA1-3	AUAP1	GGCCACGCGTCGACTAGTAC	F		5’ Abridged Universal Amplification Primer, supplied with 5’ RACE System for Rapid Amplification of cDNA Ends Kit
		AUAP2	GGCCACGCGTCGACTAGTAC	R		3’ Abridged Universal Amplification Primer, supplied with 3’ RACE System for Rapid Amplification of cDNA Ends Kit
		AP	GGCCACGCGTCGACTAGTACTTTT TTTTTTTTTTTTT	R		3’ Adapter Primer for synthesis of 1st-stranded cDNA, supplied with 3’ RACE System for Rapid Amplification of cDNA Ends Kit

F/R: forward primer/reverse primer.

TABLE S4 | List of primer sequences used to detect WCLaV-1 and WCLaV-2 by RT-PCR.

Primer name	Sequences (5′-3′)	F/R	Product size (bp)
MP-1F	TCCCTTGAACTCTTTTCCAC	F	1005
MP-1R	TAACTAGCCGCTTTTCATAAG	R	
NP-1F	ACCCTCAACATCTGGTTTCT	F	902
NP-1R	AAAACACAAAGATCCCATTA	R	
MP-2F	TAGACTTCAGAACCTCAACATCC	F	644
MP-2R	AACAGACCAATCGGTGCAAG	R	
NP-2F	CTAAATTCCCAGTGAGAGCAAC	F	778
NP-2R	GCAGTTGAGAGCTTGCCCAC	R	

F/R: forward primer/reverse primer.

TABLE S5 | List of abbreviations and accession numbers for viruses used in this study.

Virus	Virus abbreviation	Accession number					
		RNA1	RNA2	RNA3	RdRp	MP	NP
European mountain ash ringspot-associated virus	EMARaV	AY563040	DQ831828	DQ831831	AAS73287	ABH05069	ABH05070
Rose rosette virus	RRV	HQ871942	HQ871945	HQ871944	ADZ54688	ADZ54691	ADZ54690
Raspberry leaf blotch virus	RLBV	FR823299	FR823302	FR823301	CBZ42024	CBZ42027	CBZ42026
High Plains wheat mosaic virus	HPWMoW	KJ939623	KJ939627	KJ939626	AIK23031	AIK23035	AIK23034
Rice stripe virus	RSV	D31879	D10979	X53563	NP_620522	NP_620519	NP_620548
Rice grassy stunt virus	RGSV	AB009656	AB010378	AB010377	BAA32246	BAA22534	BAA22532
Rift Valley fever virus	RVFV	DQ375403.1	—	DQ380151.1	ABD51507	—	ABD38733
SFTS virus	SFTSV	HM745930	—	HM745932	ADZ04470	—	ADZ04472
Uukuniemi virus	UUKV	D10759	—	M33551	BAA01590	—	AAA47958
Sandfly fever Naples virus	SFNV	X68414	—	X53794	CAA48478	—	CAA37803
Punta Toro virus	PuTV	KP272028	—	KP272030	AKF42419	—	YP_009142918
Salehabad virus	SALV	KJ939330	—	KJ939332	AJK91618	—	AJK91621
Candiru virus	CDUV	HM119407	—	HM119409	AEA30057	—	AEA30071
Bujaru virus	BUJV	HM566164	—	HM566166	AEL29657	—	AEL29659
Tomato spotted wilt virus	TSWV	D10066	S48091	D00645	NP_049362	YP_009225	NP_049361
Watermelon bud necrosis virus	WBNV	GU735408	GU584185	GU584184	ADW23570	ADD83167	ADD83166
Watermelon silver mottle virus	WSMoV	AF133128	U75379	U78734	AAG43276	AAB41722	AAB36956
Impatiens necrotic spot virus	INSV	X93218	M74904	X66972	—	AAA46241	CAA47383
Groundnut bud necrosis virus	GBNV	AF025538	U42555	U27809	AAB94085	AAC55520	AAB04145
Groundnut ringspot virus	GRSV	KT972590	—	KT972594	AMZ00269	—	AMZ00273
Iris yellow spot virus	IYSV	FJ623474	AF214014	AF001387	ACM89280	AAL62022	AAB61923
Polygonum ringspot virus	PolRSV	KJ541746	KJ541745	KJ541744	AHZ45965	AHZ45963	AHZ45961

Hantaan virus	HTAV	X55901	—	M14626	CAA39394	—	AAA43837
Bunyamwera virus	BUNV	X14383	—	D00353	CAA32553	—	BAA00261
Crimean-Congo hemorrhagic fever virus	CCHFV	U88410	—	AY389508	AAQ90157	—	AAB48501
Citrus psorosis virus	CPsV	NC_006314.1	NC_006315.1	NC_006316.1	YP_089661	YP_089663	YP_089664
Lettuce ring necrosis virus	LRNV	NC_006051	NC_006052	NC_006053	YP_053236	YP_053238	YP_053239
Blueberry mosaic associated virus	BIMaV	KJ704366	KJ704367	KJ704368	AIF28241	AIF28243	AIF28244
Mirafiori lettuce big-vein virus	MLBVV	AF525933	AF525934	AF525935	AAN60447	AAN60448	AAN60449
Lettuce big-vein associated virus	LBVaV	AB075039.1	—	AB114138.1	BAC16226	—	BAD36830
Lettuce necrotic yellows virus	LNYV	AJ867584.2	—	AJ867584.2	CAI30426	—	CAI30421
Potato yellow dwarf virus	PYDV	GU734660.1	GU734660.1	GU734660.1	ADE45274	ADE45271	ADE45268

—: Not applicable.
